# Pin1, a new player in the fate of HIF-1α degradation: an hypothetical mechanism inside vascular damage as Alzheimer’s disease risk factor

**DOI:** 10.3389/fncel.2014.00001

**Published:** 2014-01-17

**Authors:** Elena Lonati, Anna Brambilla, Chiara Milani, Massimo Masserini, Paola Palestini, Alessandra Bulbarelli

**Affiliations:** Department of Health Science, University of Milano-BicoccaMonza (MI), Italy

**Keywords:** Alzheimer’s disease, cerebrovascular deficiencies, hippocampal neurons, Pin1, HIF-1α, GSK-3β, oxygen glucose deprivation

## Abstract

Aetiology of neurodegenerative mechanisms underlying Alzheimer’s disease (AD) are still under elucidation. The contribution of cerebrovascular deficiencies (such as cerebral ischemia/stroke) has been strongly endorsed in recent years. Reduction of blood supply leading to hypoxic condition is known to activate cellular responses mainly controlled by hypoxia-inducible transcription factor-1 (HIF-1). Thus alterations of oxygen responsive HIF-1α subunit in the central nervous system may contribute to the cognitive decline, especially influencing mechanisms associated to amyloid precursor protein (APP) amyloidogenic metabolism. Although HIF-1α protein level is known to be regulated by von Hippel-Lindau (VHL) ubiquitin-proteasome system, it has been recently suggested that glycogen synthase kinase-3β (Gsk-3β) promotes a VHL-independent HIF-1α degradation. Here we provide evidences that in rat primary hippocampal cell cultures, HIF-1α degradation might be mediated by a synergic action of Gsk-3β and peptidyl-prolyl *cis/trans* isomerase (Pin1). In post-ischemic conditions, such as those mimicked with oxygen glucose deprivation (OGD), HIF-1α protein level increases remaining unexpectedly high for long time after normal condition restoration jointly with the increase of lactate dehydrogenase (LDH) and β-secretase 1 (BACE1) protein expression (70 and 140% respectively). Interestingly the Pin1 activity decreases about 40–60% and Pin1^S16^ inhibitory phosphorylation significantly increases, indicating that Pin1 binding to its substrate and enzymatic activity are reduced by treatment. Co-immunoprecipitation experiments demonstrate that HIF-1α/Pin1 in normoxia are associated, and that in presence of specific Pin1 and Gsk-3β inhibitors their interaction is reduced in parallel to an increase of HIF-1α protein level. Thus we suggest that in post-OGD neurons the high level of HIF-1α might be due to Pin1 binding ability and activity reduction which affects HIF-1α degradation: an event that may highlight the relevance of ischemia/HIF-1α as a risk factor in AD pathogenesis.

## Introduction

Alzheimer’s disease (AD) is a multifactor neurodegenerative pathology affecting the elderly population. The pathogenesis of sporadic late-onset AD has not been identified, but further studies support that cerebral ischemia/stroke significantly increases AD risk (Kalaria, 2000; Zhang et al., 2007). Indeed, it has been suggested that cerebral hypoperfusion causing neuronal damage in vulnerable brain areas (Koistinaho and Koistinaho, 2005; Zhang et al., 2007) may serve as a basis for some cases of dementia after stroke (Ogunshola and Antoniou, 2009).

At the molecular level, a large percentage of hypoxic responses are controlled by hypoxia-inducible transcription factor-1 (HIF-1; Webb et al., 2009) whose involvement in neurodegenerative disorders is becoming widely accepted, although its role may greatly depend on whether it is the cause or the consequence in disease progression (Ogunshola and Antoniou, 2009; Bulbarelli et al., 2012).

HIF-1 is a heterodimeric protein composed of a constitutively expressed HIF-1β subunit and oxygen-regulated HIF-1α subunit (Wang et al., 2006). Under hypoxic conditions HIF-1α is stabilized, and in complex to the constitutive HIF-1β subunit induces the transcription of a plethora of genes (Semenza, 2003), whereas in normoxic conditions it is rapidly subjected to proteasomal degradation (Webb et al., 2009). HIF-1α degradation system displays enormous plasticity since it can be induced by hydroxylation and phosphorylation events either alone or in combination (Flügel et al., 2007). Indeed, although HIF-1α degradation mainly depends on two prolyl hydroxylation allowing the binding with tumor suppressor von Hippel-Lindau protein (VHL; Salceda and Caro, 1997; Sonenberg and Gingras, 1998; Semenza, 2010), evidences show that glycogen synthase kinase-3β (Gsk-3β), by phosphorylation of Ser551, Thr555, and Ser589 residues in HIF-1α oxygen degradation domain (ODD), can promote its ubiquitination and proteasomal degradation in a VHL-independent manner (Flügel et al., 2007).

In addition, during a brief hypoxic event, the HIF-1α protein stabilization needs the Akt-induced inhibitory phosphorylation of Gsk-3β on Ser9 residue (Mottet et al., 2003). A growing number of reports showed that both hypoxic and non hypoxic stimuli appear to promote HIF-1α stabilization by means of PI3K/Akt pathway (Zhong et al., 2000; Zundel et al., 2000; Hirota and Semenza, 2001; Li et al., 2008) in a cell and tissue specific way (such as in cortical neurons; Zhang et al., 2009).

The Gsk-3β-mediated degradation of HIF-1α implies a scenario similar to that of c-myc and cyclin E (Yeh et al., 2004, 2006) which upon phosphorylation by the kinase and isomerase-mediated conformational change, are ubiquitinated and degraded in the proteasome. Gsk-3β can indeed cooperate synergistically with the peptidyl-prolyl *cis/trans* isomerase (Pin1) in ubiquitination of a wide range of proteins (Liou et al., 2011). Gsk-3β as a proline-directed kinase can selectively phosphorylate Ser/Thr-Pro residues allowing the Pin1 substrate recognition and their *cis* to *trans* isomerization. The *cis* or *trans* conformation of phospho-Ser/Thr-Pro motif, as recently suggested, could be a crucial determinant in regulating protein degradation (Liou et al., 2011) in view of the fact that the ubiquitin E3 ligase complex might have a structural preference for phosphorylated substrates via a *trans* conformation. Pin1-mediated conformational change in phospho-Ser/Thr-Pro motifs, hence, represents a novel molecular switch in a large number of biological processes. Therefore, Pin1 is tightly regulated by multiple levels (Lu et al., 2002), and alterations in its functionality often lead to several pathologies, included cancer and neurodegeneration (such as AD; Lu et al., 2007). In pathological conditions, the Pin1 ability to interact with downstream substrates is inhibited by phosphorylation of Ser16 residue in its binding domain (Eckerdt et al., 2005; Lonati et al., 2011) while oxidative modification in the catalytic domain can abolish the enzymatic activity of isomerase (Butterfield et al., 2006a,b).

Although recent studies highlight indirect link between HIF-1α regulation/activity and Pin1 overexpression in breast cancer (Kim et al., 2008) and in prostate cancer (Yuan et al., 2011), little is known about the relationship of this two proteins in neuronal tissues, under physiological or pathological conditions. Consistent with that we asked whether Pin1 might participate in HIF-1α modulation under normoxic and post-ischemic conditions, such as those mimicked after oxygen glucose deprivation (OGD) treatment, where HIF-1α protein levels are carefully regulated and Pin1 activity might be altered.

Here we show that, in rat primary hippocampal cultures, Pin1 interacts with HIF-1α, and catalyzing its isomerization plays a central role in Gsk-3β-mediated proteasomal degradation of the transcription factor. Moreover in neurons subjected to OGD, Pin1 binding and activity interestingly are partially inhibited affecting HIF-1α ubiquitination and protein level.

Considering that recent studies performed in the central nervous system highlight the pathophysiological relevance of hypoxia/HIF-1 pathways regulation of β-secretase 1 (BACE1) expression and amyloid precursor protein (APP) amyloidogenic metabolism (Zhang et al., 2007; Bulbarelli et al., 2012), alterations in HIF-1α protein levels/degradation pathway may contribute to the cognitive decline and dementia in AD patients influencing the disease course.

## Materials and methods

### Materials

All commercial chemicals were of the highest available grade: Sprague-Dawley rats were from Charles-River Laboratories (Lecco, Italy). The 5% CO_2_: 95% N_2_ gas cylinder was from Sapio, Monza, Italy. Complete protease inhibitor cocktail was from Roche Diagnostics S.p.A (Milano, Italy). Hydroxy-1,4-naphtoquinone (juglone), Lactacystin, 1-β-D arabinofuranosylcytosine (Ara-C), lithium chloride solution, SB-216763 Gsk-3 inhibitor, solutions for electrophoresis were from Sigma Chemical Co. (Milano, Italy). All the stock solutions for cell culture were from Euroclone (Celbio Milano, Italy). Gibco Neurobasal medium (NBM) and B27 supplement, Dynabeads® protein G, sodium dodecyl sulphate (SDS) NuPAGE reagents (4–12% Bis-Tris gel; sample buffer; running buffer), Novex Sharp Protein Standard, anti-Tau was from Life Technologies (Milano, Italy).

Anti-Pin1, anti P-Pin1^S16^ and anti-Ubiquitin (PD41) were from Cell Signaling (Beverly, USA). Anti-HIF-1α and anti-lactate dehydrogenase (LDH) antibodies were from Abcam (Cambridge Science Park, UK). Anti P-Ser/Thr-Pro (MPM2) and Anti-Pin1 for immunoprecipitation antibodies were from Millipore S.p.A (Milano, Italy). Anti-BACE1 antibody was from Santa Cruz Biotechnology Inc. (Santa Cruz, CA, USA). Secondary HRP-conjugated antibodies and ECL SuperSignal detection kit were from Pierce (Rockford, IL, USA). Anti-Actin and anti P-Tau^T231^ antibodies were from Sigma Chemical Co (Milano, Italy).

### Cell culture

Hippocampal neurons cultures were prepared from E18-E19 rat hippocampi as previously described, with minor modifications (Brewer et al., 1993; Bulbarelli et al., 2009). Neurons were plated on polylysine coated dishes (60 mm diameter, 5 × 10^5^ cells/dish). The medium for cell culture was NBM containing 2% B27 supplement and 12.5 nM glutamate. After 72 h in culture, half of the cell medium was replaced with NBM w/o glutamate and supplemented with 1-β-D arabinofuranosylcytosine (Ara-C) (5 µM final concentration) to prevent glial proliferation. Cells were maintained at 37°C, 5% CO_2_ for 8 days before treatment. All experiments were carried out in accordance with the guidelines established by the European Community Council and were approved by the Italian Ministry of Health (DL 116/92). Adequate measures were always taken to minimize animal pain or discomfort.

### Oxygen and glucose deprivation treatment

Primary hippocampal neurons were subjected to OGD as previously described (Bulbarelli et al., 2012). Briefly, culture medium was replaced by through exchange with a glucose-free balanced salt solution (BSS; NaCl 130 mM, KCl 5.5 mM, CaCl_2_ 1.8 mM, MgCl_2_ 1 mM, HEPES 20 mM). Then cells incubated in a hypoxia chamber (Billups–Rothenberg, Del Mar, CA) saturated for 10 min with 5% CO_2_: 95% N_2_ were sealed at 37°C for 3 h. After OGD, cells were supplemented with the restoration solution: glucose (final concentration 5 mM) and B27 (final concentration 2%) in NBM.

Cells were maintained in normal culture conditions (37°C in a 5% CO_2_ atmosphere) for different times of restoration: one hour (R 1 h) and overnight (R o/n) after the reestablishment of normoxia. Untreated hippocampal cells were incubated in NBM supplemented with 2% B27 in a 5% CO_2_ atmosphere.

### Treatments with inhibitors

5-Hydroxy-1,4-naphtoquinone (juglone) was dissolved in dimethyl sulfoxide (DMSO) to obtain a 10 mM stock solution and used as Pin1 catalytic activity inhibitor, accordingly to previous studies (Chao et al., 2001; Galas et al., 2006). Hippocampal cells were incubated with a 10 µM juglone final concentration for 8 h as already described (Bulbarelli et al., 2009), or with 1 µM lactacystin, an inhibitor of ubiquitin-proteasome degradation system, for 16 h (Cazzaniga et al., 2007). According to literature Gsk-3 kinase activity was inhibited by means of lithium chloride administrated to hippocampal cells at 10 mM final concentration for 1 h (Stambolic et al., 1996) or SB-216763 administrated at 10 µM (Facci et al., 2003) for 3 h accordingly to experimental indications obtained in our cellular model.

### Immunoprecipitation

Cells subjected or not to OGD or to inhibitor treatments for different times, were rinsed twice with phosphate-buffered saline (PBS) and harvested by scraping with a rubber policeman after lysis with non denaturising buffer (1% Nonidet P-40, 50 mM Tris-HCl, pH 7.4, 150 mM NaCl) in presence of complete protease inhibitor cocktail and phosphatase inhibitors (2 mM Na orthovanadate, 1 mM Na fluoride, 1 mM Na pyrophosphate). Then, immunoprecipitation of the endogenous HIF-1α, Pin1 or P-Ser/Thr-Pro (MPM2) proteins was performed from 1 mg of total proteins using anti-HIF-1α (1:100), anti-Pin1 (1:100), anti-MPM2 (1:100) and Dynabeads protein G according to the manufacturer’s instruction. Immunoprecipitated proteins were eluted in lauryl-dodecyl sulphate (LDS) lysis buffer with reducing agent (Life Technologies) and boiled for 10 min at 70°C before analysis on NuPAGE Bis-Tris 4–12% polyacrylamide gels.

### SDS-PAGE electrophoresis and immunoblotting

Samples were obtained after lysis with denaturising buffer (2% SDS lysis, 50 mM Tris-HCl, pH 6.8 plus protease inhibitor cocktail and phosphatase inhibitors). The total protein amount was evaluated by means of Bicinchoninic acid assay and 25 µg of each sample were subjected to on SDS-polyacrylamide gel electrophoresis (PAGE) on 12.5% polyacrylamide gels. For immunoprecipitation, samples obtained were loaded on NuPAGE Bis-Tris 4–12% gels. After SDS-PAGE electrophoresis samples were transferred to a nitrocellulose membrane (Amersham, GE Healthcare Europe GmbH, Milano, Italy) and proteins revealed by Ponceau staining (Sigma Chemical Co., Milano, Italy). Membranes were blocked in TBS-Tween 0.1% buffer containing 5% non-fat milk or TBS-Tween 0.2% buffer containing 3% bovine serum albumin, and probed with specific antibodies in TBS-T buffer containing 5% non-fat milk or 5–3% bovine serum albumin according to manufacturer’s instructions. Immunoblotting was performed using anti-Pin1 (1:1000), anti-P-Pin1^S16^ (1:1000), anti-HIF-1α (1:1000), anti-ubiquitin (PD41) (1:1000), anti LDH (1:2000), anti-BACE1 (1:1000), anti-P-Ser/Thr-Pro (MPM2) (1:1000), anti-P-Tau^T231^ (1:5000), anti-Tau (1:1000) and anti-β-actin (1:1500). Immunoreactive proteins were revealed by enhanced chemiluminescence (ECL) and semi-quantitatively estimated by a KODAK image station 2000R. Normalization was carried out with respect to the amount β-actin in the same sample.

### PPIase assay

Pin1 activity was measured according to the methods of Janowski et al. (1997) and Hennig et al. (1998) with slight modifications as follows (Janowski et al., 1997; Hennig et al., 1998). Stock solutions for the protease-free assay was prepared as described: the phosphorylated substrate Ac-AA(pS)PR-pNA was dissolved in 0.47 M LiCl/trifluoroethanol (anhydrous) at 30 mg/ml concentration; trypsin protease was dissolved in 35 mM HEPES, pH 7.8 at 50 mg/ml concentration.

For a typical measurement, to determine the PPIase activity originating from Pin1, the sample buffer (35 mM HEPES, pH 7.5) was incubated in a temperature-controlled cuvette holder at 4°C. Then Ac-AA(pS)PR-pNA (0.02 mg/ml) was added and the reaction was started by the injection of trypsin (0.1 mg/ml). After the initial burst phase where all trans peptides were cleaved, protein samples obtained after lysis, as described for immunoprecipitation, was added to the reaction. The absorption at 390 nM, which detects the formation of free *p*-nitroanilide (pNA), was monitored using a Beckman Coulter DU 800 spectrophotometer. The enzymatic activity of Pin1 in the sample (unit/mL) was calculated as follows: absorbance (OD) * total volume/9620 M^-1^ cm^-1^ (extinction coefficient *ϵ*) * volume of sample in mL * 1 cm (*d*). Normalization was carried out with respect to total protein in the same sample.

Unit/mL=((ΔAbs/min⁡)∗(V tot in mL))/(ε∗(V of sample in mL)∗d)

The measured activity was normalized to the total protein content of the lysate.

### Statistical analysis

All data are expressed as mean ± SEM of three separate experiments performed in triplicate. The differences were calculated by means of Student’s *t*-test. A *p* value <0.05 was considered to be statistically significant.

## Results

### Effects of the oxygen and glucose deprivation on HIF-1α protein expression, pin1 phosphorylation and activity in hippocampal neurons cultures

Hippocampal cells were subjected to OGD for 3 h. Following the treatment, we investigated the HIF-1α protein levels after 1 hour (R 1 h) and overnight (R o/n) of normal oxygen and glucidic conditions restore. As already demonstrated by our group (Bulbarelli et al., 2012), hippocampal neuron viability is not affected by the OGD treatment.

**Figure 1:**
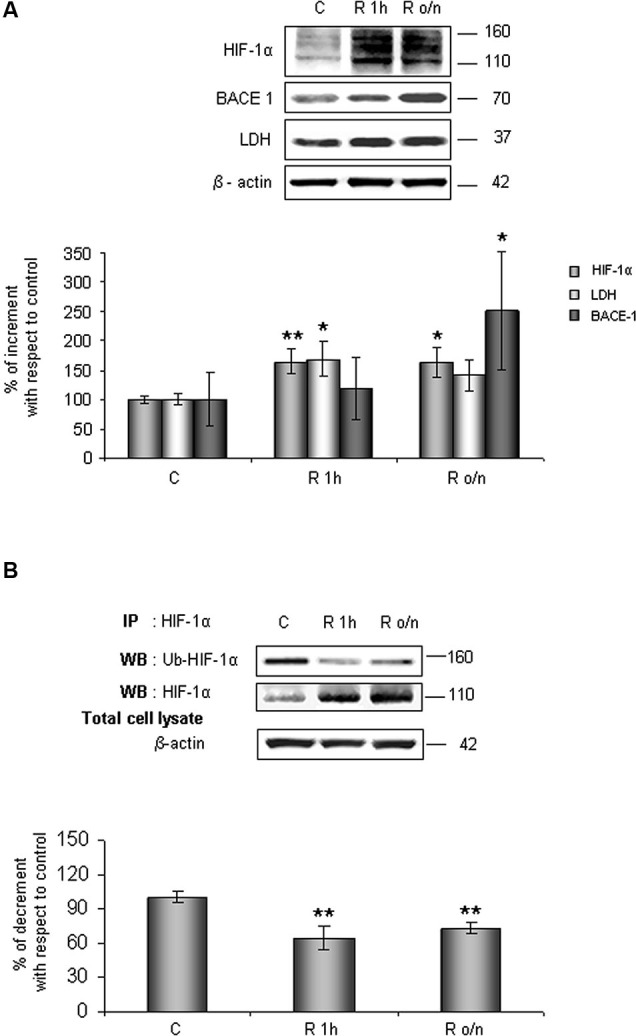
**In post-OGD conditions HIF-1α protein levels increases while its ubiquitination state decreases**. Cells were subjected to OGD treatment and restoration of normoxic and glucidic conditions for different times (R 1 h, R o/n). **(A)** Cells were collected and lysed with 2% SDS lysis buffer. Equal amounts of samples (as protein content) were subjected to SDS-PAGE and western blot analysis. HIF-1α, LDH, BACE1 levels were detected by specific antibodies, revealed by enhanced chemiluminescence (ECL) and semi-quantitatively determined by a KODAK image station 2000R. Anti-β-actin antibody was employed to confirm equal protein loading in the different lanes. Results obtained are shown on the bar-graphs. Normalization was carried out with respect to the β-actin amount in the same sample. **(B)** HIF-1α ubiquitination state was evaluated after immunoprecipitation using western blot analysis. Cells were collected and lysed in non denaturising NP40 buffer. Western blot analysis performed on immunoprecipitated proteins showed bands corresponding to ubiquitinated HIF-1α protein (140 kDa), HIF-1α protein (110 kDa). Proteins were detected by specific antibodies (anti-HIF-1α; anti-ubiquitin), revealed by ECL and semi-quantitatively determined by a KODAK image station 2000R and the results obtained are shown on the bar-graphs. IP, immunoprecipitation; WB, western blot. Data represent the mean ± SEM of three separate experiments performed in triplicate. Statistical significance is obtained with Student’s *t*-test in comparison with controls. * *p* < 0.05, ** *p* < 0.01.

As shown in Figure 1A, HIF-1α protein level increased at R 1 h (60%) remaining significantly higher than control up to R o/n. Concomitantly, we detected that HIF-1α ubiquitination state was decreased about 40% at R 1 h almost unvarying at R o/n (Figure 1B).

Relatively to HIF-1α expression increase after normal condition restoration (post-OGD) we examined lactate dehydrogenase (LDH) and BACE1 protein expression, two proteins whose *genes* are under HIF-1α transcriptional regulation. We observed that LDH expression increased about 70% already at R 1 h, slightly decreasing during overnight, while BACE1 protein level significantly increased during o/n, reaching 150% of increment (Figure 1A).

**Figure 2:**
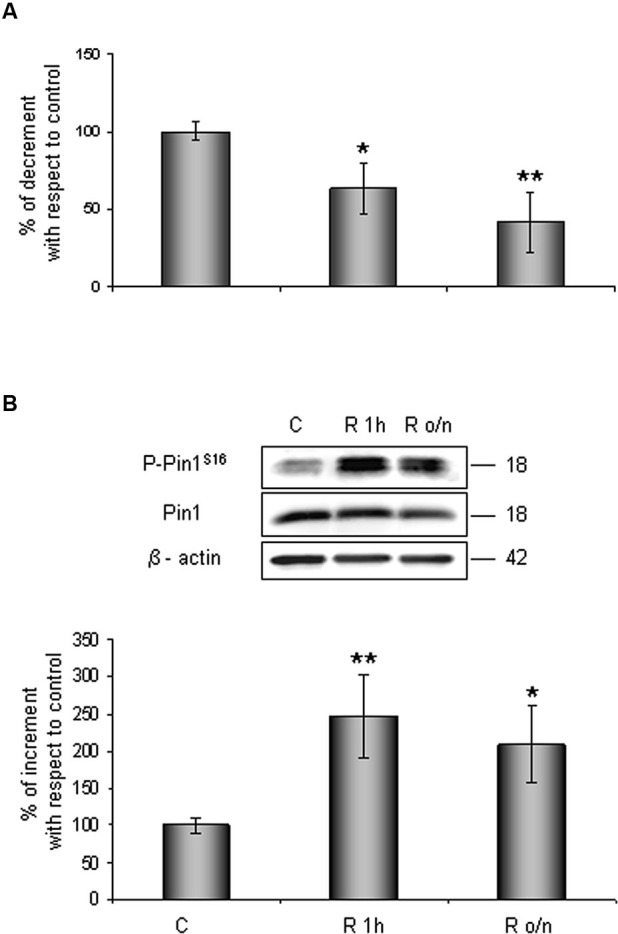
**Pin1 inhibition in post-OGD conditions. (A)** PPIase activity decreases during restoration of normal cultures conditions following OGD. Cells were lysed and collected in non denaturising NP40 buffer at R 1 h and R o/n. Pin1 isomerase activity against Ac-AA(pS)PR-pNA substrate were measured as described in materials and methods. The measured activity was normalized to the total protein concentration of the lysate. **(B)** Pin1^S16^ phosphorylation increases in response to OGD treatment. Cells were lysed in the presence of 2% SDS lysis buffer after OGD treatment and restoration of normoxic and glucidic conditions for different times (R 1 h, R o/n). Total protein content were subjected to SDS-PAGE and Western blot analysis. P-Pin1^S16^ and total Pin1 were detected by specific antibodies and revealed by ECL, and semi-quantitatively determined by a KODAK image station 2000R. Anti-β-actin antibody was employed to confirm equal protein loading in the different lanes. Results obtained are shown on the bar-graphs. The amount of P-Pin1^S16^ correspond to phospho-protein level on Pin1 total protein. Normalization was carried out with respect to the β-actin amount in the same sample. Data represent the mean ± SEM of three separate experiments performed in triplicate. Statistical significance is obtained with Student’s *t*-test in comparison with controls. * *p* < 0.05, ** *p* < 0.01.

Contemporary we observed that Pin1 activity was reduced in a time dependent manner of about 40% at R 1 h, decreasing till 60% during overnight (Figure 2A). In addition, in the same conditions we assessed Pin1^S16^ phosphorylation (a state that inhibits Pin1 binding to its substrate): the isomerase was significantly phosphorylated at R 1 h (150%) slightly decreasing at R o/n (110%; Figure 2B).

### Phosphorylation of HIF-1α Ser/Thr-Pro motifs for specific Pin1 recognition and interaction

**Figure 3:**
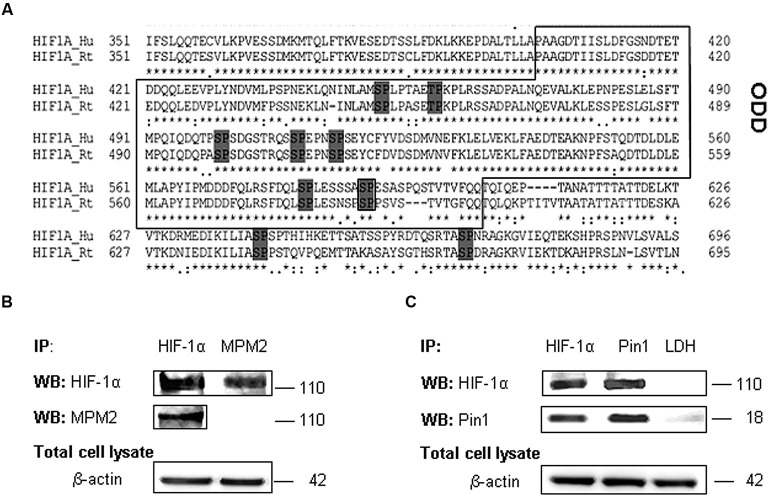
**HIF-1α phosphorylation on PSer/Thr-Pro motifs and interaction with Pin1. (A)** Alignment of amino acid sequences of the HIF-1α oxygen degradation domain (ODD) (aa 402-604) from HIF-1α human and rat homologues. PSer/Thr-Pro motifs are highlighted in dark gray. **(B)** Cells were collected and lysed in non denaturising NP40 buffer. Immunoprecipitated HIF-1α is recognized by MPM2 antibody, vice versa among immunoprecipitated MPM2 epitopes, HIF-1α antibody detects the corresponding protein. **(C)** Co-immunoprecipitation of endogenous HIF-1α and Pin1 proteins in primary rat hippocampal cells lysed in non denaturising NP40 buffer. Immunoprecipitation with LDH antibody was performed as negative control. Western blotting analysis performed on immunoprecipitated proteins showed a 110 kDa and a 18 kDa band corresponding to HIF-1α and Pin1 respectively. Proteins detected by specific antibodies, were revealed by ECL. IP, immunoprecipitation; WB, western blot; MPM2, antibody against phosphorylated Ser/Thr-Pro motifs.

We examined the amino acid HIF-1α protein sequence to evaluate the presence of conserved Ser/Thr-Pro motifs potentially recognizable by Pin1 when phosphorylated (Flügel et al., 2007), by means of the alignment of human (Q16665) (Iyer et al., 1998) and rat (O35800) HIF-1α protein sequence (Kietzmann et al., 2001; Align tool EXPASY). As shown the analyses revealed a number of these motifs in HIF-1α sequence. In particular, the Gsk-3β target residue Ser589 precedes a proline in one of the above mentioned Ser/Thr-Pro motifs (Figure 3A).

Therefore in order to find one or more HIF-1α phosphorylated Ser/Thr-Pro motifs (PSer/Thr-Pro), we employed the monoclonal antibody MPM2, which specifically recognizes the PSer/Thr-Pro motifs. Immunoprecipitated HIF-1α protein was identified by MPM2 antibody, confirming that HIF-1α protein contains PSer/Thr-Pro motifs under normal culture conditions. On the other hand, among proteins immunoprecipitated with MPM2, HIF-1α was identified by a specific antibody (Figure 3B).

Given that this phosphorylation might allow Pin1 interaction with its hypothetic substrate, we therefore tested in our neuronal model whether HIF-1α associates with Pin1 by co-immunoprecipitation of endogenous HIF-1α or Pin1. As shown in Figure 3C, HIF-1α was detected among proteins immunoprecipitated by the anti-Pin1 antibody as well Pin1 was detected in the protein complex immunoprecipitated by the anti HIF-1α antibody suggesting that HIF-1α and Pin1 might interact each other. Immunoprecipitation with LDH antibody was performed as negative control.

### Pin1 inhibition affects HIF-1α degradation pathway

**Figure 4:**
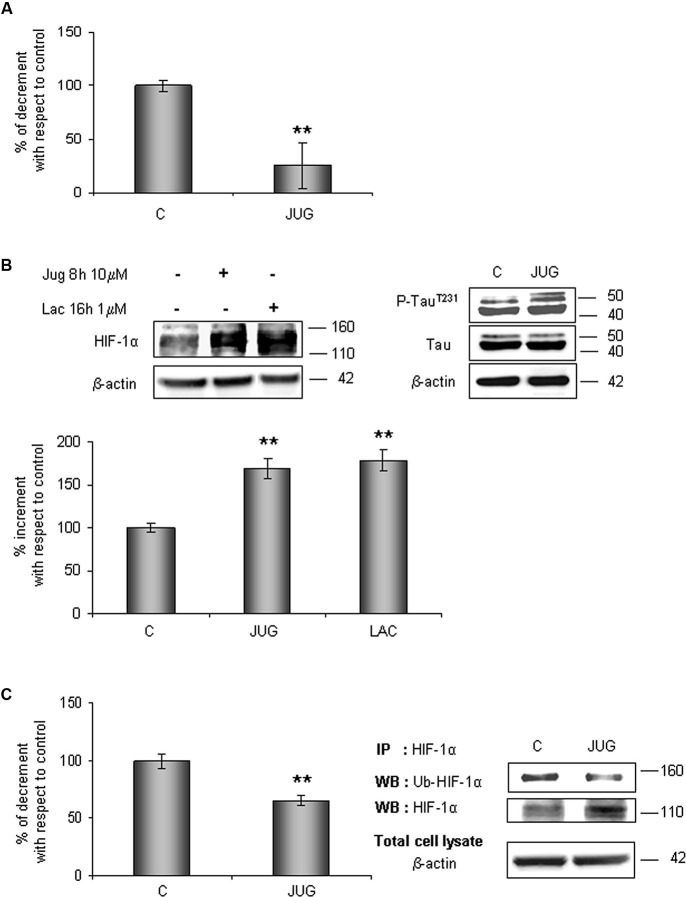
**HIF-1α protein levels increases in parallel to a decrease of its ubiquitination state after juglone (jug) treatment**. Cells were treated or not with juglone 10 µM for 8 h. **(A)** PPIase activity decreases in cells subjected to juglone treatment. Cells were lysed and collected in non denaturising NP40 buffer and Pin1 isomerase activity against Ac-AA(pS)PR-pNA substrate were measured as described in Section materials and methods. The measured activity was normalized to the total protein concentration of the lysate. **(B)** Neurons were treated or not with juglone or lactacystin (lac) 1 µM for 16 h. Equal amounts of total cell lysates (in the presence of 2% SDS) were subjected to SDS-PAGE and western blot analysis. HIF-1a, Tau, P-Tau^T231^ levels were detected by specific antibodies, revealed by ECL and semi-quantitatively determined by a KODAK image station 2000R. HIF-1α results obtained are shown on the bar-graphs. Anti-β-actin antibody was employed to confirm equal protein loading in the different lanes, normalization was carried out with respect to the β-actin amount in the same sample. **(C)** HIF-1α ubiquitination state decreases after juglone treatment. Western blot analysis showed a 110 kDa and 140 kDa bands corresponding to HIF-1α and to ubiquitinated HIF-1α protein respectively. Proteins were detected by specific antibodies (anti-HIF-1α; anti-ubiquitin), revealed by ECL and semi-quantitatively determined by a KODAK image station 2000R. Results obtained are shown on the bar-graphs. IP, immunoprecipitation; WB, western blot. Data represent the mean ± SEM of 3 separate experiments performed in triplicate. Statistical significance is obtained with Student’s *t*-test in comparison with controls. * *p* < 0.05, ** *p* < 0.01.

In order to understand whether Pin1 plays a role in HIF-1α ubiquitination and the following degradation, hippocampal cells were treated for 8 h with 10 µM juglone, an irreversible inhibitor of Pin1 catalytic activity. As expected, juglone treatment triggered a strong inhibition of Pin1 isomerase activity (almost 75%; Figure 4A).

The effect of Pin1 inhibition was firstly studied in the whole lysate. As shown in Figure 4B, HIF-1α protein level increased of about 70% with respect to control level. Since it is known that Pin1 promotes Tau dephosphorylation (Bulbarelli et al., 2009), a more convincing evidence of the Pin1 inhibition was obtained evaluating Tau phosphorylation on residue Thr231. As expected we observed an increase in Tau phosphorylation.

In parallel, to evaluate the proteasomal degradation of HIF-1α in our cellular model, cells were incubated for 16 h with 1 µM lactacystin an irreversible proteasome inhibitor, revealing HIF-1α protein increment of about 80%. Data obtained are comparable to ones detected after juglone, suggesting that Pin1 might be engaged in the HIF-1α degradation. Contemporary, immunoprecipitated HIF-1α resulted about 35% less ubiquitinated than in untreated cells (Figure 4C).

### Gsk-3β inhibition influences Pin1/HIF-1α association

To assess the involvement of Gsk-3β activity in phosphorylation of Ser/Thr-Pro motifs, hippocampal neurons were incubated in presence of 10 mM LiCl for 1 h, or in presence of 10 µM SB-216763 for 3 h. Then we performed HIF-1α protein immunoprecipitation to analyse phosphorylation state in Ser/Thr-Pro motifs and the amount of Pin1 co-immunoprecipitated.

**Figure 5:**
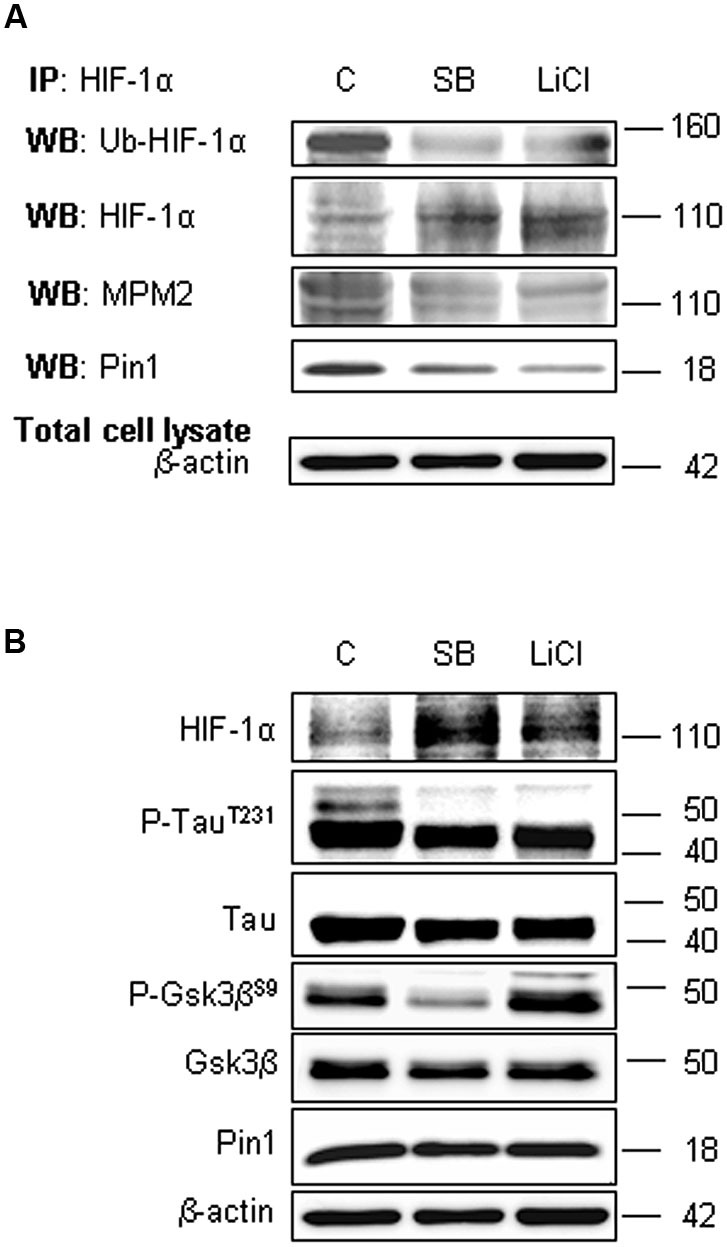
**Lithium Chloride (LiCl) or SB-216763 treatment inhibits Gsk-3β leading to a decrease in HIF-1α phosphorylation on PSer/Thr-Pro motifs and in HIF-1α/Pin1 interaction**. Cells were treated or not with LiCl 10 mM for 1 h or SB-216763 10 µM for 3 h. **(A)** Western blot analysis performed on HIF-1α immunoprecipitated protein revealed a decrease of the ubiquitination state (anti-ubiquitin), of Ser/Thr-Pro motifs phosphorylation (anti-MPM2) and of Pin1 co-immunoprecipitation (anti-Pin1). **(B)** Equal amounts of total cell lysates (in the presence of 2% SDS) were subjected to SDS-PAGE and Western blot analysis. HIF-1α, Tau, P-Tau^T231^, Gsk-3β and P-Gsk-3β^S9^ and Pin1 levels were detected by specific antibodies and revealed by ECL. Tau^T231^ phosphoryalation as well known Gsk-3β target was assessed to evaluate the kinase inhibition. Anti-β-actin antibody was employed to confirm equal protein loading in the different lanes. IP, immunoprecipitation; WB, western blot; MPM2, antibody against phosphorylated Ser/Thr-Pro motifs.

As shown in Figure 5A, phosphorylation of Ser/Thr-Pro motifs was greatly reduced in cells treated with the inhibitors, concomitantly to a considerable decrease in the amount of co-immunoprecipitated Pin1, although total Pin1 amount resulted almost unchanged (Figure 5B). In addition, we observed the HIF-1α ubiquitination decrease and a slight enhance of protein level both in the immunoprecipitated fractions (Figure 5A) and in the total lysates (Figure 5B), indicating a possible reduction in the HIF-1α protein degradation.

In order to confirm the Gsk-3β inhibition, Figure 5B shows the increase of Gsk-3β inhibitory phosphorylation on Ser9 after LiCl treatment, accordingly to Martin et al. (2009). Moreover, inhibitory effect on Gsk-3β activity was assessed evaluating Tau phosphorylation on Thr231 residue, a well known target of kinase activity (Figure 5B; Lin et al., 2007). As expected, after treatment with both the inhibitors, Tau phosphorylation decreased.

### Effects of the oxygen and glucose deprivation on Gsk-3β phosphorylation and HIF-1α/Pin1 association

**Figure 6:**
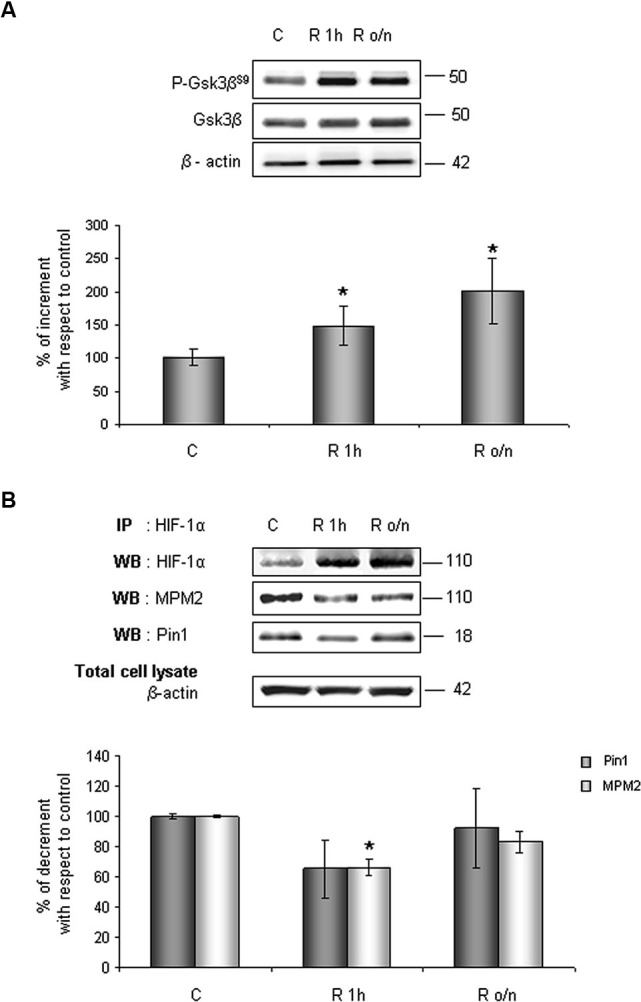
**In post-OGD conditions P-Gsk-3β^S9^ levels increases**. Cells were subjected to OGD treatment and restoration of normoxic and glucidic conditions for different times (R 1 h, R o/n). **(A)** Cells were collected and lysed with 2% SDS lysis buffer. Equal amounts of samples (as protein content) were subjected to SDS-PAGE and western blot analysis. Gsk-3β and P-Gsk-3β^S9^ levels were detected by specific antibodies, revealed by ECL and semi-quantitatively determined by a KODAK image station 2000R. Anti-β-actin antibody was employed to confirm equal protein loading in the different lanes. Results obtained are shown on the bar-graphs. The amount of P-Gsk-3β^S9^ correspond to phospho-protein level on Gsk-3β total protein. Normalization was carried out with respect to the β-actin amount in the same sample. **(B)** HIF-1α phosphorylation on Ser/Thr-Pro motifs and HIF-1α/Pin1 interaction was evaluated after HIF-1α immunoprecipitation using western blot analysis. Cells were collected and lysed in non denaturising NP40 buffer. Western blot analysis performed on immunoprecipitated proteins showed bands corresponding to Pin1 protein (18 kDa), HIF-1α protein and HIF-1α phosphorylated on Ser/Thr-Pro motifs (110 kDa). Proteins were detected by specific antibodies (anti-HIF-1α; anti-MPM2; anti-Pin1), revealed by ECL and semi-quantitatively determined by a KODAK image station 2000R and the results obtained are shown on the bar-graphs. IP, immunoprecipitation; WB, western blot. Data represent the mean ± SEM of three separate experiments performed in triplicate. Statistical significance is obtained with Student’s *t*-test in comparison with controls. * *p* < 0.05, ** *p* < 0.01.

Considering results above, to investigate whether HIF-1α ubiquitination decrease in post-OGD conditions might correlate to Gsk-3β inhibition, the P-Gsk-3β^S9^ levels were evaluated. As shown in Figure 6A, Gsk-3β^S9^ inhibitory phosphorylation increased about 40% at R 1 h and duplicated at R o/n, while total Gsk-3β protein resulted unchanged during the treatment. Concomitantly we detected a significant decrease in Ser/Thr-Pro motifs phosphorylation, revealed by anti-MPM2, on the immunoprecipitated HIF-1α protein at R 1 h (Figure 6B). At R o/n we observed a slightly increase of P-Ser/Thr-Pro motifs that however did not reach control levels (Figure 6B). Moreover, we also evaluated the co-immunoprecipitated Pin1 amount: a considerable decrease in HIF-1α/Pin1 association was revealed at R 1 h, while the Pin1 amount at R o/n was quite equivalent to the control (Figure 6B).

## Discussion

Coexistence of ischemic and neurodegenerative pathology seems to have a deep impact on the expression of the dementia, suggesting common mechanisms interactions. Cerebrovascular diseases such as ischemia leading to cerebral blood flows reduction (hypoperfusion) might indeed initiate and/or accelerate the neurodegeneration cascade *via* amyloid deposition and synaptic neuronal dysfunction (Iadecola, 2010; Kalaria and Ihara, 2012). Depletion of oxygen and glucose sources typical of brain ischemia (Brouns and De Deyn, 2009), results in the activation of highly heterogeneous phenomena in which one of the key component is the transcription factor HIF-1. Therefore protein levels of its hypoxia responsive α subunit (HIF-1α) are finely regulated by degradation through ubiquitin-proteasome system that may be induced by hydroxylation (VHL-mediated) and as recently suggested by phosphorylation (Gsk-3β-mediated; Flügel et al., 2007, 2012). Here we propose that, in hippocampal cellular model, the Pin1 plays an important role in Gsk-3β-mediated HIF-1α ubiquitination/degradation pathway.

In cortical neurons, as already demonstrated, HIF-1α protein levels are maintained higher for long time after reperfusion following OGD by PI3K/Akt signaling (Zhang et al., 2009). Since the PI3K/Akt pathway is known to inhibit Gsk-3β and to regulate HIF-1α in a cell type specific manner (Mottet et al., 2003), we speculate that in neuronal cells HIF-1α degradation might be also regulated by the Gsk-3β pathway working in parallel or in alternative to the well known VHL-mediated mechanism.

Gsk-3β regulates the ubiquitination state of several targets in cooperation with Pin1 (Liou et al., 2011), that mediating prolyl *cis/trans* isomerization can influence the stability of its substrates via phosphorylation-dependent ubiquitin-mediated proteolysis both under physiological and pathological conditions; accordingly, Pin1 might interact and catalyze the isomerization of HIF-1α phosphorylated by the kinase under physiological conditions. Interestingly, here we show, for the first time in neurons, that OGD treatment results in a Pin1 activity reduction, an event that in turn leads to HIF-1α protein levels increase. Indeed, although the transcription factor should be immediately degraded in normoxic conditions by VHL-mediated pathway restoring control levels (Semenza, 2010), in hippocampal cells high levels of HIF-1α protein are detectable at R 1 h and maintained for long time (even up to overnight) after normal oxygen and glucidic conditions restore following OGD. Moreover, HIF-1α high levels are accompanied by a significant decrease of protein ubiquitination state, indicating an impairment in degradation pathway.

Concerning to Pin1, OGD triggers a partial inhibition of its enzymatic activity and also increases Ser16 residue phosphorylation in the binding domain. Therefore, on one side Ser16 phosphorylation may inhibit Pin1 function in recognizing and binding its substrates, on the other side Pin1 isomerization activity might be diminished by oxidative modification occurring in the catalytic domain (Lu et al., 2002; Lonati et al., 2011). Indeed, as already known oxidative stress might reduce Pin1 function (Butterfield et al., 2006a,b).

Considering data above, we suppose that Pin1 partial inhibition may directly affect the HIF-1α degradation pathway, thus we evaluated if Pin1/HIF-1α interaction might be required for HIF-1α ubiquitination.

Firstly, after exploring in HIF-1α amino acid sequence the presence of motifs constituted by serine/threonine residues preceding a proline (Ser/Thr-Pro) that, when phosphorylated, allows Pin1 to specifically recognise its substrates, we found these phosphorylated motifs in HIF-1α protein, employing the anti-MPM2 antibody (which specifically recognizes the PSer/Thr-Pro motifs). Given that this evidence pinpoints the transcription factor as an hypothetic target for Pin1 binding, we provided the evidence of HIF-1α/Pin1 association by co-immunoprecipitation, indicating that the transcription factor and the isomerase may interact each other.

Afterwards, we evaluated the HIF-1α level expression, inhibiting Pin1 catalytic activity by means of juglone treatment. Since we had already established that juglone-mediated Pin1 inhibition leads to Tau^T231^ phosphorylation increase (Bulbarelli et al., 2009), here we show Tau phosphorylation augment after juglone as positive control of the treatment. Under conditions in which Pin1 enzymatic activity is highly repressed, data obtained showed that HIF-1α levels strongly increase in parallel with a mild decrease of its ubiquitination state, demonstrating that Pin1 affects protein ubiquitination and degradation. Interestingly the HIF-1α levels observed after juglone treatment were comparable to that observed in proteasome inhibited cells suggesting that in our cellular model, Pin1 might play a main role in the fate of the transcription factor. Taking into account that HIF-1α and Pin1 co-immunoprecipitate, it is plausible that the enzyme catalyzes HIF-1α peptydil-prolyl conformational isomerization.

As Liou and colleagues reported (Liou et al., 2011), Pin1 can cooperate synergistically with Gsk-3β in ubiquitination of a wide range of proteins, therefore the role of this kinase in Ser-Thr/Pro phosphorylation and the consequent interaction between Pin1 and HIF-1α have been investigated.

Among the several Gsk-3β phosphorylation consensus motifs in HIF-1α amino acid sequence individuated by Flügel et al. (2007), we observed that the Ser589 is neighboring to a proline constituting a Ser-Thr/Pro motif, which phosphorylated might be the consensus for Pin1 recognition. Therefore we speculate that this sequence could be significant in the HIF-1α stability.

To understand whether decreasing HIF-1α phosphorylation levels might affect Pin1/HIF-1α interaction, we employed two different Gsk-3β activity inhibitors: the well known LiCl and the more specific SB-216763. Since LiCl treatment induces Gsk-3β inhibitory phosphorylation on Ser9 (Martin et al., 2009), here we confirme the kinase inhibition showing an increase in Ser9 phosphorylation. On the contrary, SB-216763 induces the dephosphorylation of Gsk-3β at Ser9 (Jaeger et al., 2013), nevertheless inhibits Gsk-3β activity in an ATP competitive manner (Cross et al., 2001).

**Figure 7:**
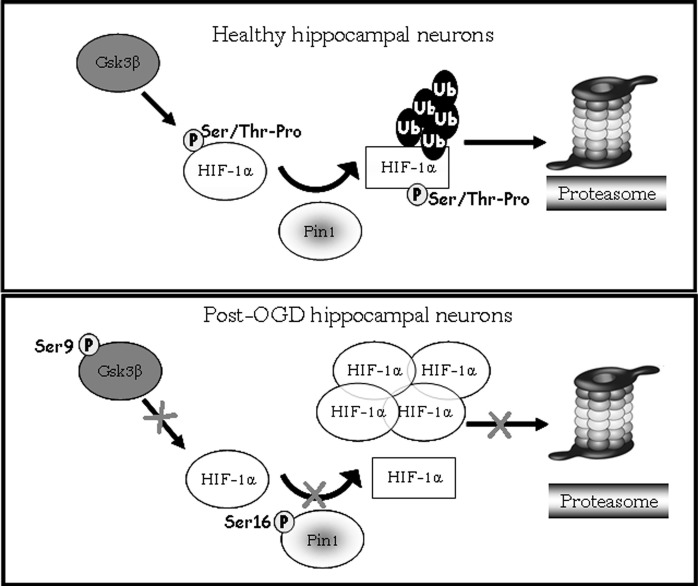
**Model showing HIF-1α degradation pathway mediated by Pin1/Gsk-3β**. In healthy hippocampal neurons Gsk-3β phosphorylates the HIF-1α transcription factor on Ser/Thr-Pro motif potentially recognizible by Pin1. After phosphorylation Pin1 is able to recognize and bind the *cis* form of HIF-1α (circle form), and catalyzes its isomerization to *trans* conformation (square). The E3 ligase complex having a structural preference for phosphorylated substrates in *trans* conformation to favors ubiquitination and the consequent degradation of the transcription factor *via* proteasome. In post-OGD neurons, instead, Gsk-3β is inhibited by phosphorylation on Ser9, therefore HIF-1α is not phosphorylated on Ser/Thr-Pro motif resulting unrecognizable by Pin1. Moreover Pin1 itself is inhibited by phosphorylation on Ser16, and its enzymatic activity is blocked leading to loss of HIF-1α *cis/trans* isomerization. HIF-1α is not more ubiquitinated and degradated via proteasome, accumulating in its *cis* conformation.

Interestingly, data obtained after the treatment with Gsk-3β activity inhibitors showed: (i) a strong reduction of phosphorylated Ser/Thr-Pro motifs of HIF-1α protein; (ii) a considerable decrease of co-immunoprecipitated Pin1 amount; (iii) an higher HIF-1α protein level with respect to control. The reduction of HIF-1α phosphorylated motifs accompanied by protein levels increase, give reason for Pin1 binding decrease, suggesting that Pin1 might be involved in the Gsk-3β/HIF-1α stability regulation independent from the VHL pathway (Flügel et al., 2007). Indeed, Flügel et al. (2007) suggest that the regulation of HIF-1α degradation is not limited by the presence of oxygen, resulting therefore independent of HIF-1α hydroxylation and VHL-E3 ligase complex. Moreover, the F-box protein Fbw7, an E3 ubiquitin ligase, has been recently identified as novel critical component in HIF-1α degradation that is recruited to HIF-1α after its phosphorylation by Gsk-3β (Flügel et al., 2012): in a wider framework we can speculate that Pin1 might be involved in the Gsk-3β/Fbw7 HIF-1α degradation system. Taken together our experiments indicate for the first time that Pin1 interacts with HIF-1α in order to regulate the transcription factor protein levels. Therefore the decrease of Pin1 ability to bind and isomerize PSer/Thr-Pro motif might correlate with high levels of HIF-1α observed in post-OGD conditions (see a schematic representation in Figure 7). To deepen the OGD effect on HIF-1α and Pin1 interaction we analyzed Gsk-3β^S9^, HIF-1α Ser/Thr-Pro motif phosphorylation and HIF-1α/Pin1 association. Interestingly, here we show that, Gsk-3β^S9^ inhibitory phosphorylation increased at R 1 h, correlating both to the Ser/Thr-Pro motifs phosphorylation decrease and HIF-1α/Pin1 association reduction. *Vice versa* at R o/n we observed a slightly increase of P-Ser/Thr-Pro motifs that however did not reach control levels and the co-immunoprecipitated Pin1 amount was quite equivalent to the control. This observation apparently in contrast with the HIF-1α increase might be explained by the fact that although Pin1 might be able to bind the transcription factor, since Pin1^S16^ phosphorylation decreased, the isomerase resulted enzymatically inhibited (see the PPIase assay in Figure 2). Hence, we hypothesize that in post-OGD hippocampal neurons, Gsk-3β and Pin1 inhibition lead to a decrease of HIF-1α ubiquitination affecting the consequent degradation of the transcription factor. Since, unconventional VHL-independent HIF-1α degradation pathway involving RACK1 has been already described (Isaacs et al., 2002; Semenza, 2010), here we propose that Pin1/Gsk-3β-mediated HIF-1α degradation may be a functionally predominant pathway in our cellular model, that instead of VHL requires the involvement of the E3 ubiquitin ligase Fbw7.

Furthermore, it has been recently proposed that Pin1 inhibits Gsk-3β activity (Ma et al., 2012) promoting Ser9 phosphorylation, however in our cellular model subjected to OGD this event seems to be independent from Pin1, deserving future experiments to deeply investigate.

For all above mentioned, under condition mimicking an ischemic event influencing Pin1 activity might be extremely detrimental causing intracellular molecular mechanisms deregulation, eventually leading to pathophysiological conditions such as in AD. In fact Pin1 has been identified as a common regulator of both Tau and APP pathologies (Lu and Zhou, 2007), regulating Tau phosphorylation levels and the NFTs formation as well as levels of Aβ peptide production (Liou et al., 2003; Pastorino et al., 2006).

Our group has recently demonstrated that after OGD the amount of peptide Aβ_42_ increases both in neurons and in cerebrovascular endothelial cells (Bulbarelli et al., 2012), probably related to the increase of HIF-1α transcriptional activity (Zhang et al., 2007).

Notably, in parallel to HIF-1α high levels we observed the increase of BACE1 protein under our experimental conditions. Hence, in our hypothesis, the alteration in Pin1-mediated HIF-1α degradation resulting from an ischemic event, might accelerate APP amyloidogenic metabolism/Aβ_42_ production in neurons, supporting the theory that cerebral hypoperfusion induces Aβ deposition. As well in AD the Pin1 deregulation might lead to a more rapid HIF-1α stability/activation inducing the expression of genes implicated in pathological intracellular mechanisms involved in vascular diseases and neurodegeneration.

In view of that there could be a pathogenic synergy between the two disease processes, stroke and AD might effectively share common risk factors (Iadecola, 2010): in this scenery the role of Pin1 in HIF-1α isomerization and degradation may outcome as central mechanism in vascular damages *vs.* AD and *vice versa*. Furthermore, considering that it has been recently demonstrated the correlation between Pin1 activity reduction and the essential hypertension (Wang et al., 2013a,b), the isomerase partial loss of function might be detrimental in multiple interrelated disease; hence the role of Pin1 in HIF-1α regulation might contribute to the morbidity of hypertension, becoming in turn a risk factor for stroke and AD.

## Conflict of interest statement

The authors declare that the research was conducted in the absence of any commercial or financial relationships that could be construed as a potential conflict of interest.

## Abbreviations

PAGE: polyacrylamide gel electrophoresis
SDS: sodium dodecyl sulphate
SEM: standard error media
TBST: Tris-buffered saline-Tween
OGD: oxygen glucose deprivation
Ara-C: 1-β-D arabinofuranosylcytosine
BSS: balanced salt solution
LDS: lauryl-dodecyl sulphate
ECL: enhanced chemiluminescence
DMSO: dimethyl sulfoxide
pNA: *p*-nitroanilide
juglone: 5-Hydroxy-1,4-naphtoquinone
APP: amyloid precursor protein
PI3K: phosphatidylinositol-3 kinase
Pin1: peptidyl-prolylcis/trans isomerase (PPIase)
HIF-1α: hypoxia-inducible transcription factor subunit-α
Gsk-3β: glycogen synthase kinase-3β
BACE1: β-secretase 1
LDH: lactate dehydrogenase
VHL: von Hippel-Lindau protein.
